# Supplementary figures and images for: Defining the Cynomolgus Macaque (Macaca fascicularis) Animal Model for Aerosolized Venezuelan Equine Encephalitis: Importance of Challenge Dose and Viral Subtype
Source: Viruses. 2023 Nov 29;15(12):2351. doi: 10.3390/v15122351 (PMC10748030; doi:10.3390/v15122351)

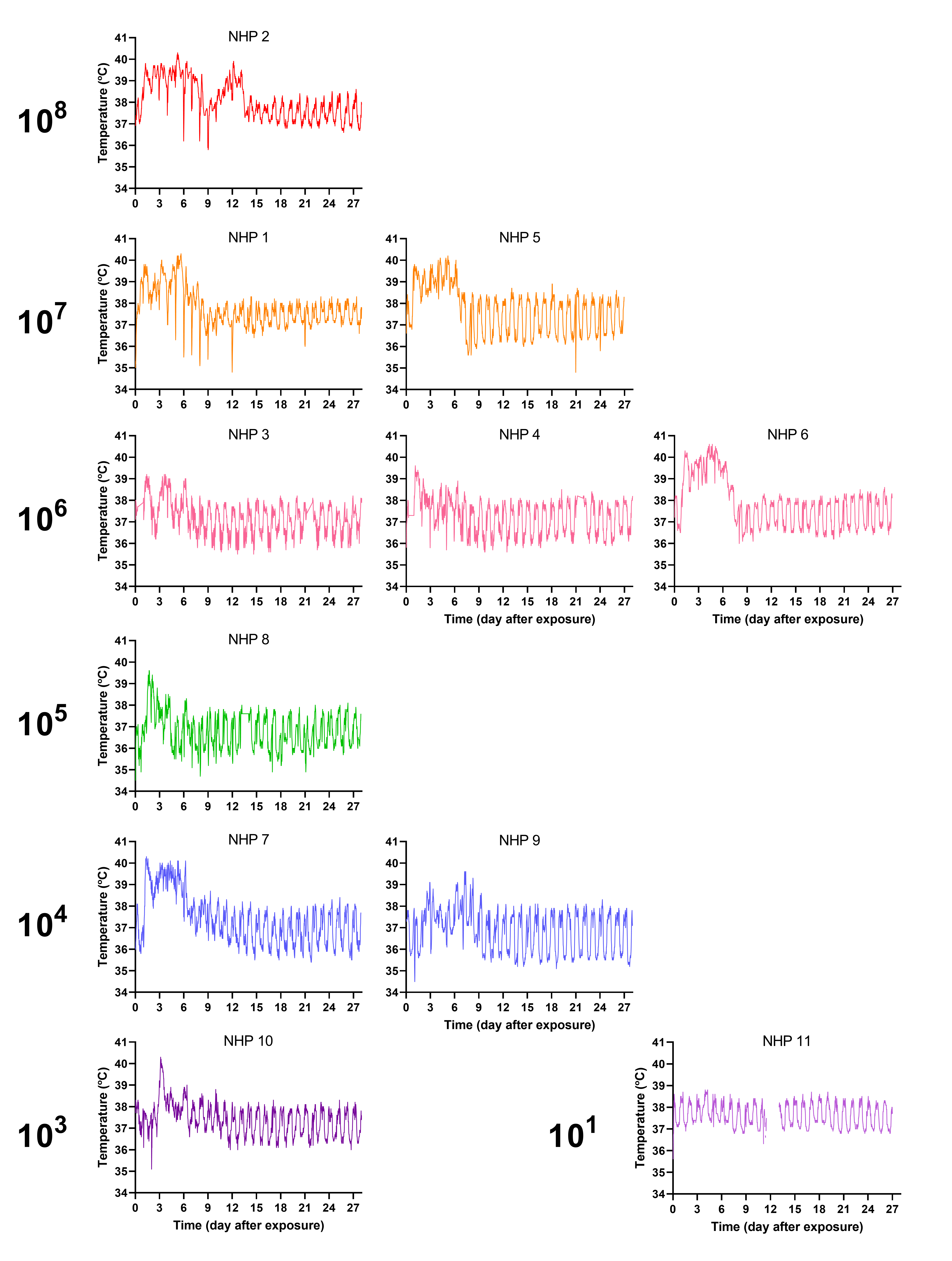

Supplement: Supplementary file 1 [file viruses-15-02351-s001.zip › Figure S1.tif]

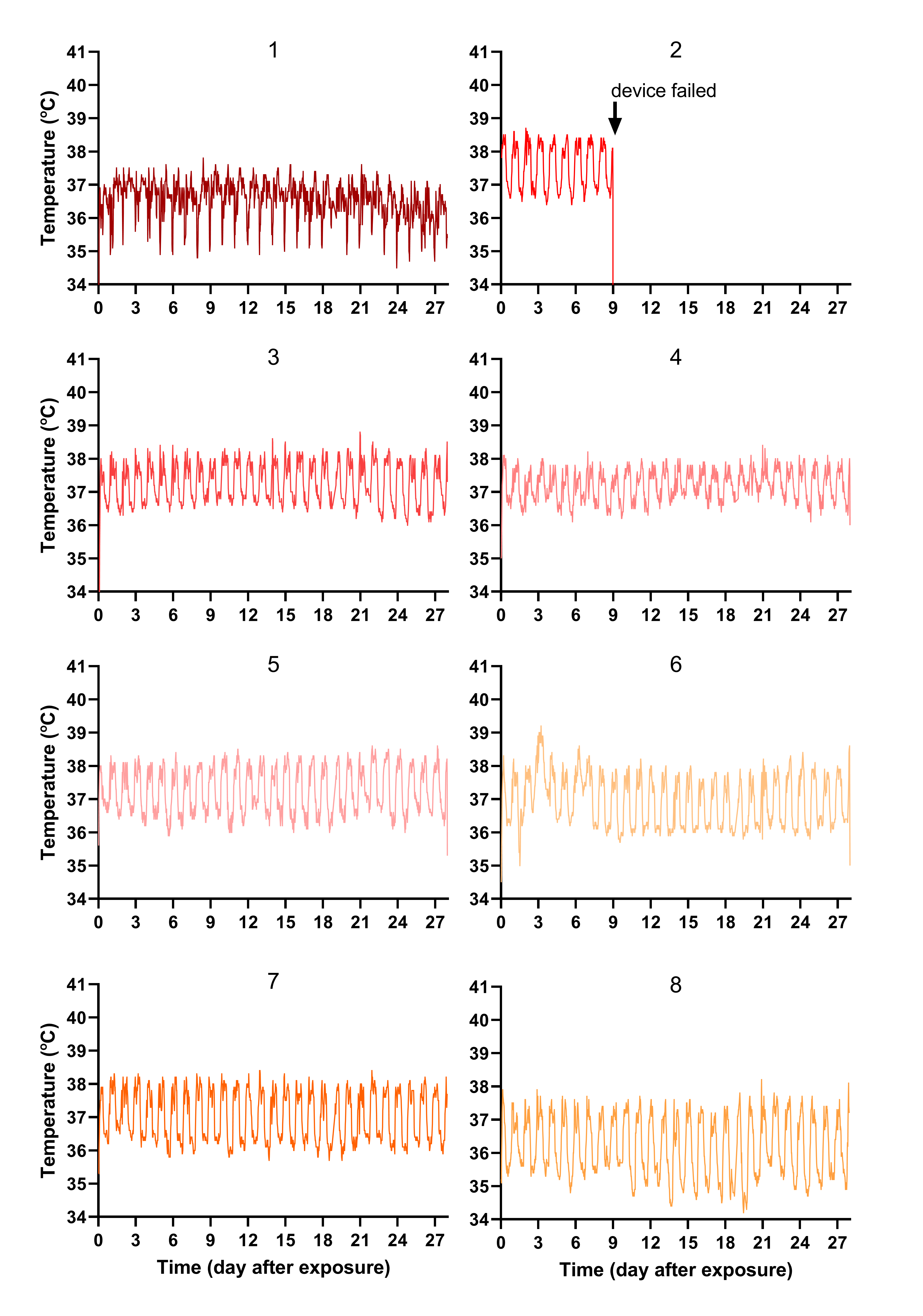

Supplement: Supplementary file 1 [file viruses-15-02351-s001.zip › Figure S2.tif]

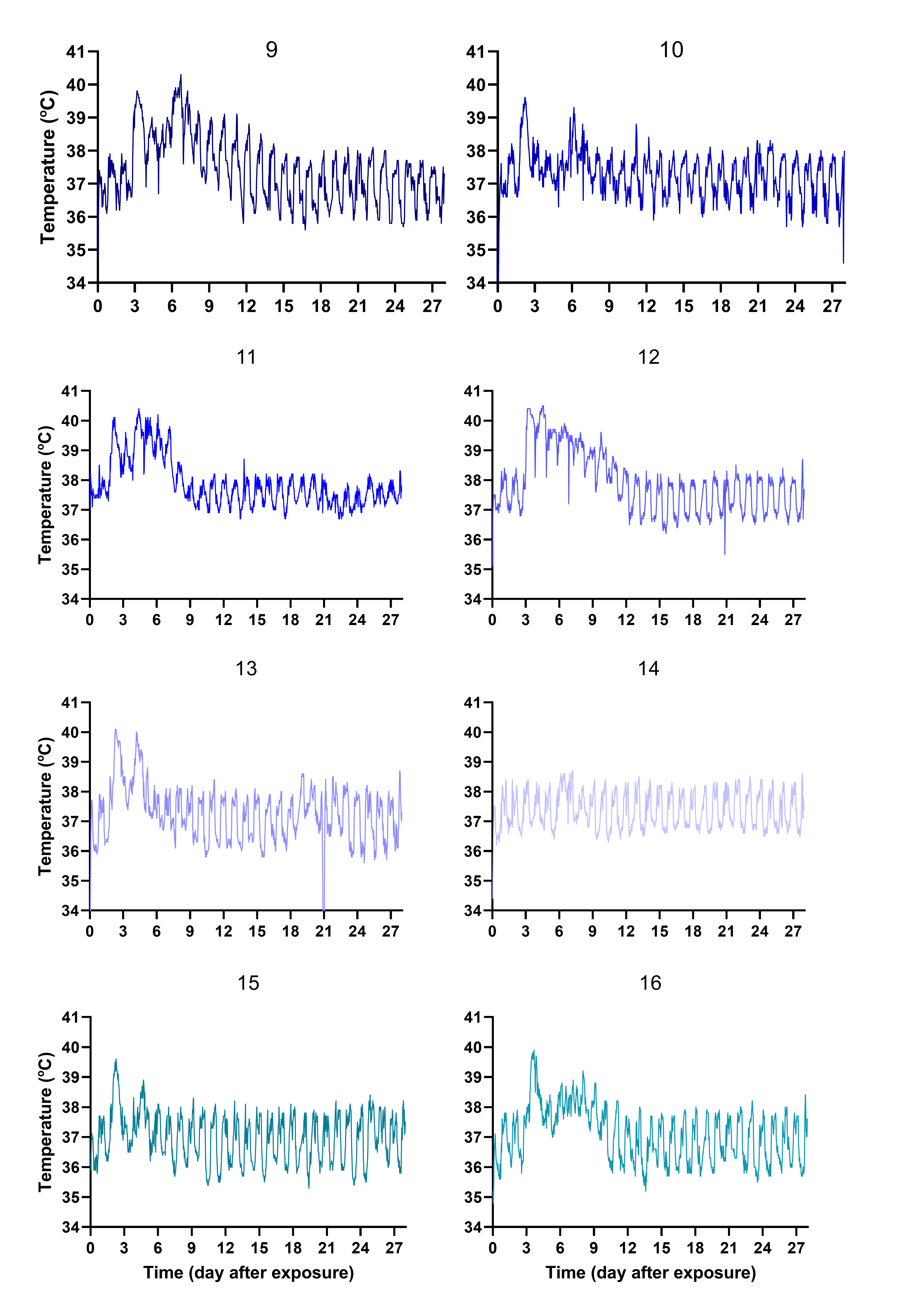

Supplement: Supplementary file 1 [file viruses-15-02351-s001.zip › Figure S3.tif]

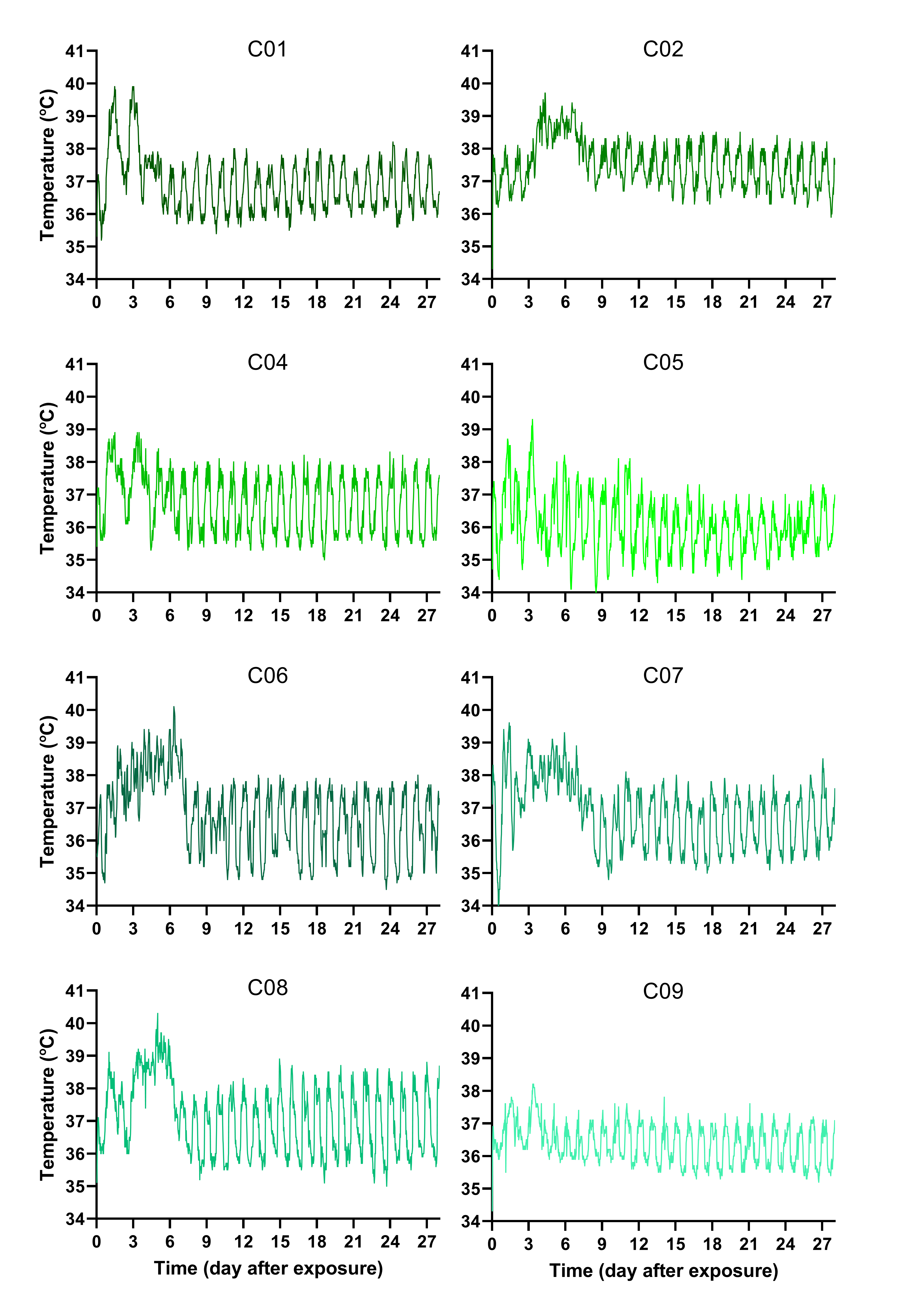

Supplement: Supplementary file 1 [file viruses-15-02351-s001.zip › Figure S4.tif]

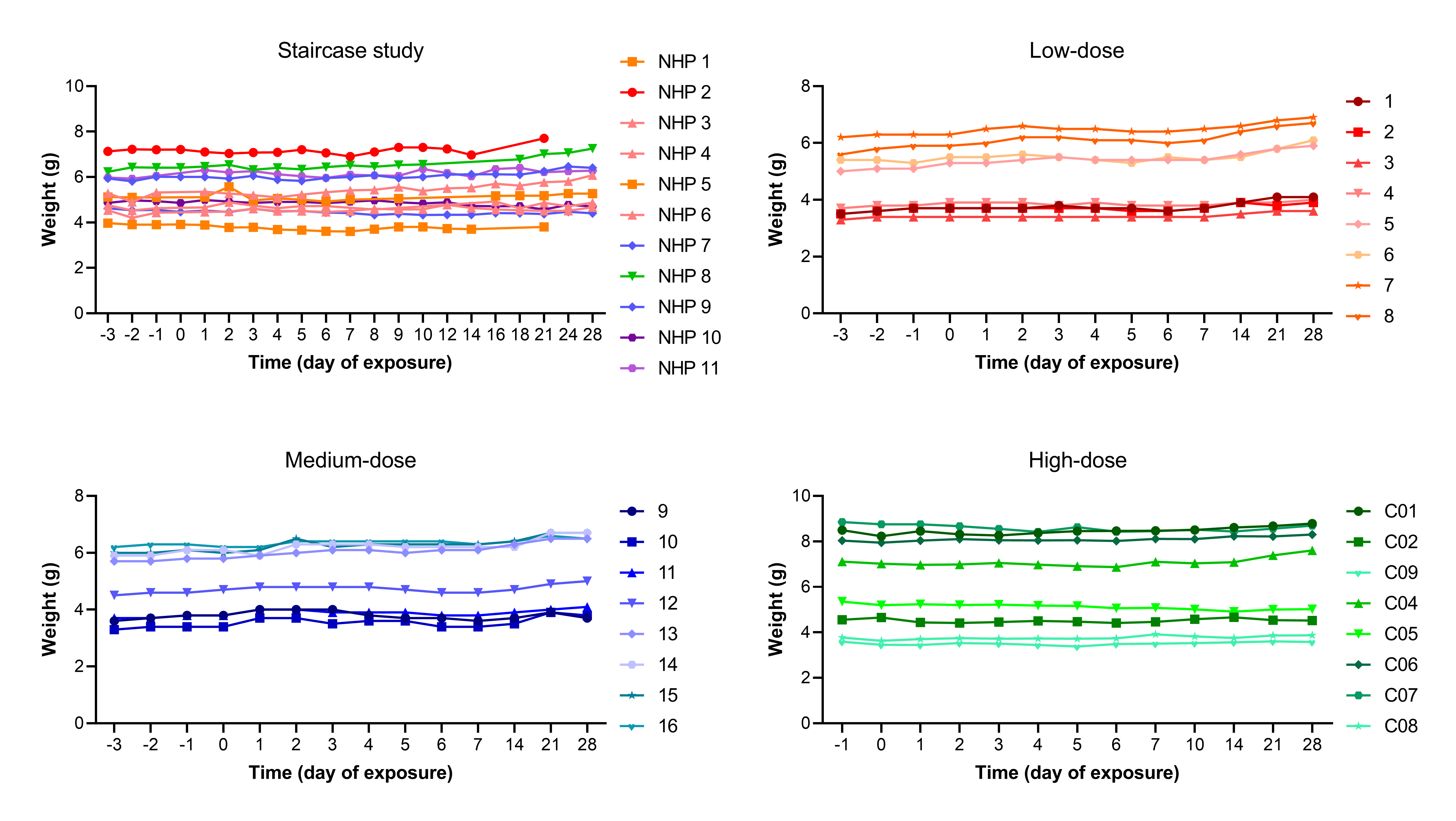

Supplement: Supplementary file 1 [file viruses-15-02351-s001.zip › Figure S5.tif]

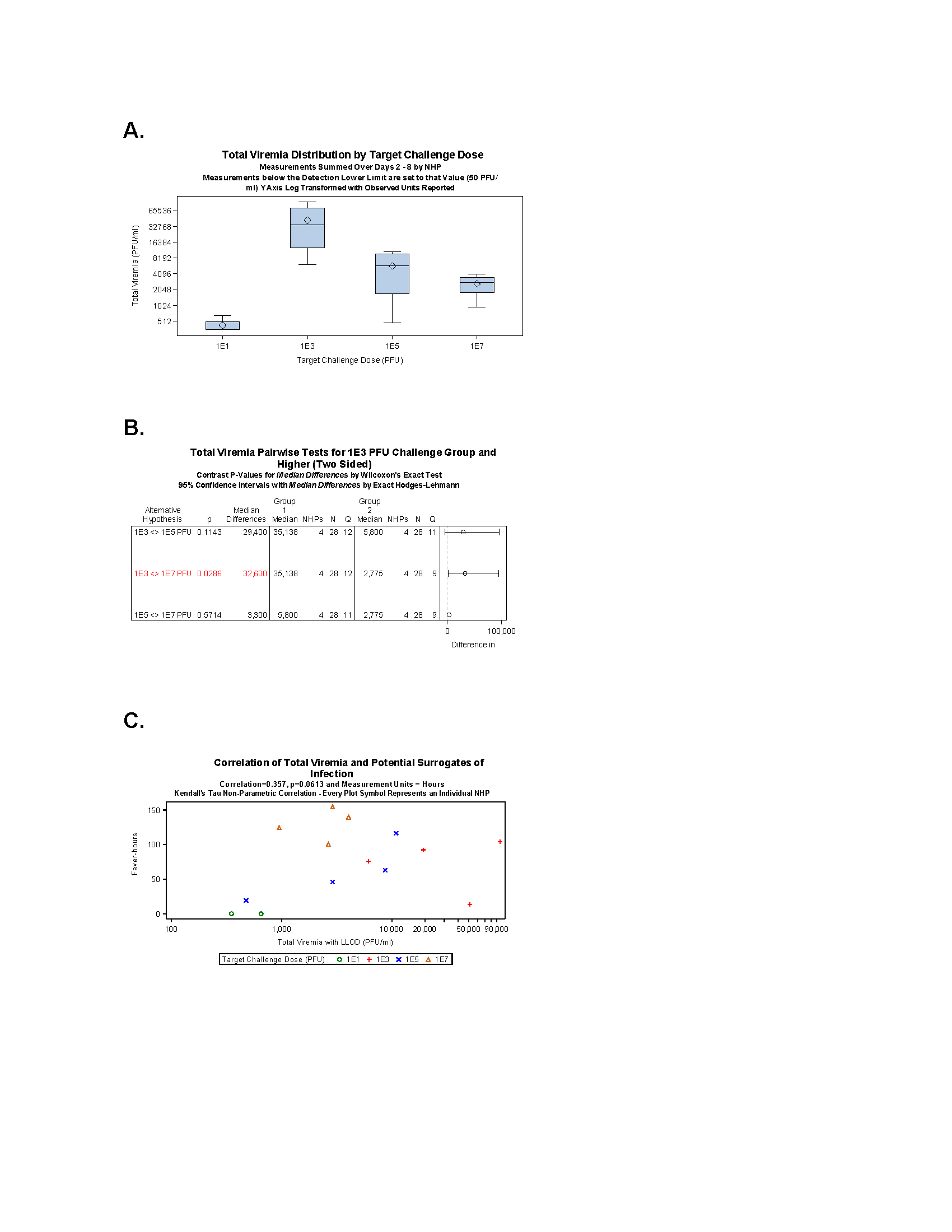

Supplement: Supplementary file 1 [file viruses-15-02351-s001.zip › Figure S6.tif]

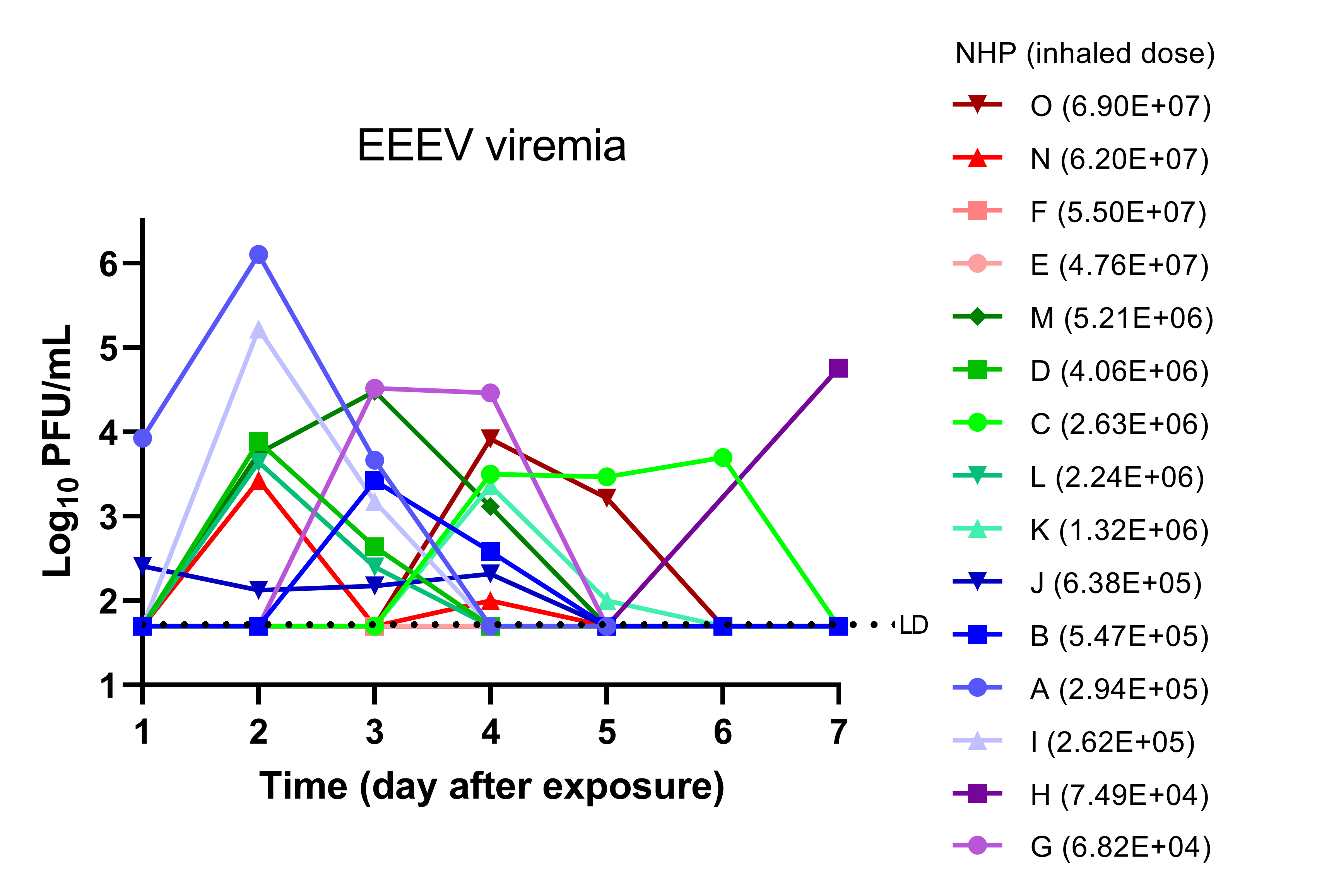

Supplement: Supplementary file 1 [file viruses-15-02351-s001.zip › Figure S7.tif]
